# Opinions on Amblyopia Treatment in Microtropia – A Questionnaire Study of Orthoptists in Scandinavia

**DOI:** 10.22599/bioj.316

**Published:** 2024-11-13

**Authors:** Jannicke Røe, Gemma Arblaster

**Affiliations:** 1 School of Allied Health Professions, Nursing and Midwifery, University of Sheffield, UK; 2 Betanien Hospital, Skien, Norway

**Keywords:** microtropia, anisometropia, diplopia, amblyopia, amblyopia treatment, Scandinavia

## Abstract

**Background:**

Microtropia is a small angle strabismus of less than or equal to ten prism diopters. It often co-exists with anisometropia, and patients may require amblyopia treatment. Diplopia following amblyopia treatment is considered rare, but older literature can advise caution when treating amblyopia in microtropia. This study aimed to explore orthoptists’ opinions on amblyopia treatment in microtropia.

**Methods:**

Orthoptists working in Scandinavia were invited to complete an online questionnaire regarding their views on amblyopia treatment. They were presented with three different clinical scenarios: 1) patient with anisometropia; 2) patient with anisometropia and microtropia with identity; and 3) patient with anisometropia and microtropia without identity.

**Results:**

The questionnaire received responses from 30 orthoptists, which were analysed. The results showed a significantly higher concern for diplopia in patients with microtropia undergoing amblyopia treatment than in patients with anisometropia. They responded that to prevent diplopia, it is more important to stop amblyopia treatment before equal visual acuity (VA) is reached in microtropia compared to anisometropia. Thus, amblyopia treatment was stopped more often in microtropia, even if VA was improving and diplopia was absent. Equal VA was perceived to be more difficult to achieve in microtropia, both with and without identity, compared to anisometropia.

**Conclusion:**

Despite more recent evidence that diplopia following amblyopia treatment is extremely rare, orthoptists working in Scandinavia reported more concerns about diplopia when treating amblyopia in microtropia than in anisometropia. Stopping amblyopia treatment in microtropia before equal VA was achieved was considered somewhat important to prevent diplopia. Orthoptists also reported that equal VA was difficult to achieve in patients with microtropia, both with and without identity. Further research would help improve the evidence and inform clinical decisions about microtropia and amblyopia treatment in microtropia.

## Background

Microtropia is a manifest strabismus of less than five degrees (Lang, 1974) or less than or equal to 10 prism dioptres (Ansons and Davis, 2014) with sensory adaptation to allow subnormal stereopsis. Features include reduced visual acuity (VA) in the deviating eye (Parks, 1969), foveal suppression of the deviating eye under binocular viewing and parafoveal fixation in the deviating eye on monocular viewing (Ansons and Davis, 2014). It has been associated with anisometropia (Lang, 1974; Parks, 1969) and heredity (Lang, 1983). Microtropia is subdivided into with and without identity. Microtropia with identity has no manifest deviation on the cover/uncover test (Ansons and Davis, 2014). Microtropia without identity has a small manifest deviation on the cover/uncover test due to a difference between the objective and subjective angle of deviation (Rowe, 2012). Monofixation syndrome (MFS) includes all patients with a foveal suppression scotoma of the non-fixating eye under binocular viewing with some fusion and stereopsis. Other MFS features variably present are history of strabismus, a unilateral macular lesion and a larger deviation on the alternate cover test than cover/uncover (Parks, 1969). MFS therefore includes both microtropia with and without identity.

Older literature suggested microtropia was a static condition with little possibility of changing the abnormal correspondence to normal correspondence with bifoveal fixation (Jampolsky, 1951; Parks, 1969; Lang, 1974). Treatment targeted at achieving normal correspondence was reported to be futile, with a risk of diplopia (Bagolini and Campos, 1983). However, recovery of microtropia to normal correspondence with bifoveal binocular single vision (BSV) following amblyopia treatment has been shown to be possible (von Noorden, 1996; Houston *et al*., 1998). Despite this, normal bifoveal BSV following amblyopia therapy is not a common outcome, even when final VA is good (Tomac, Ener and Sanac, 2002; Matsuo *et al*., 2003).

Diplopia has been reported in some patients with microtropia following amblyopia treatment (Lang, 1974; Evardhard-Halm and de Buy Wenniger-Prick, 1989), but it is considered rare (PEDIG, 2005; Newsham and O’Connor, 2016). There is little evidence supporting a higher risk of diplopia in microtropia or in older patients with amblyopia. Evidence advising caution in treating amblyopia in microtropia mostly relates to avoiding sensory treatment such as pleoptics (Campos, 1995), avoiding synoptophore training (Lang, 1974), attempting to restore bifoveal BSV and caution that patching may eliminate a central scotoma (Von Noorden, 1996).

Variable VA outcomes following amblyopia treatment in microtropia have been reported, from no improvement or slight improvement (Evardhard-Halm and de Buy Wenniger-Prick, 1989) to good VA outcomes (Houston *et al*., 1998) and equal VA with bifoveal fixation (Houston *et al*., 1998). Evardhard-Halm and de Buy Wenniger-Prick (1989) suggested that in microtropia, older age at amblyopia treatment was related to poorer VA outcomes, yet others have shown good VA improvements in older patients with microtropia (Migliorini, 2019).

Despite evidence that good VA can be achieved when treating amblyopia in microtropia and the risk of diplopia being extremely rare, there is a clinical impression that amblyopia treatment may be stopped sooner in patients with microtropia, and there is a concern about the risk of diplopia. This research study aimed to explore the opinions and clinical practice of orthoptists working in Scandinavia and, in particular, if amblyopia treatment differed when microtropia was present.

## Methods

Ethical approval was granted from the University of Sheffield (044106) as the study was undertaken as part of a master’s program. A questionnaire was distributed to members of the Scandinavian Orthoptic Association (SOA), representing Norway, Sweden, Denmark and Finland (Supplementary file 1). There were approximately 170 members at the time of the survey. The questionnaire (Google Form) included study information, consent gathering and 27 questions about amblyopia treatment. Clinical scenarios of microtropia, with and without identity, and anisometropia without microtropia were used in both older and younger patients. Anisometropia was used as the ‘control’ condition, to which the responses to questions on microtropia could be compared. The questionnaire was developed by the authors, as no suitable questionnaire existed at the time (to our knowledge). Responses were anonymous and included Likert scale (1–7) and multiple-choice answers. Text boxes were available for additional comments. Responses to the question asking for ‘country of clinical practice’ included a ‘prefer not to say’ option, due to the small numbers of orthoptists working in some countries. This allowed all responses to remain anonymous. A sample size calculation (Gpower) for a within-factors repeated measures ANOVA showed that a minimum of 12 responses were required to reach a power of 95% (medium effect size 0.5). More responses would add further information and strength to the results.

The questionnaire was available for 8 weeks. One reminder e-mail was sent out. Anonymous responses were collated in a spreadsheet and analysed using SPSS (version 26). A repeated measures ANOVA was performed using the Likert responses as the dependent variables for anisometropia, microtropia with identity and microtropia without identity. To explore the difference between the factors and control the familywise error rate, a post hoc pairwise comparison with Bonferroni adjustment was performed. Where the distribution was considered not normal, additional non-parametric analysis was conducted.

## Results

Thirty-one responses to the questionnaire were received with no missing data. Responses from one participant were removed as they were considered anomalous. It was possible they had not understood the questionnaire or the scale direction. The results of the remaining respondents are presented (n = 30). Respondents were from Norway (n = 12), Sweden (n = 7), Denmark (n = 4) and seven chose ‘not to say’ their country of practice. The amount of orthoptic experience of the respondents was mean 19.5 years (range 3–40 years).

### Diagnosing microtropia

Most orthoptists used multiple clinical tests to diagnose microtropia, including the cover test (n = 30), stereotests (n = 28), VA (n = 24), sensory fusion tests (n = 21), 4 prism dioptre (PD) prism reflex test (n = 24), measurement of refractive error (n = 19), fixation assessment using a visuoscope (n = 14) and assessment of motor fusion (n = 7).

### Concern about diplopia

**Figure 1:**
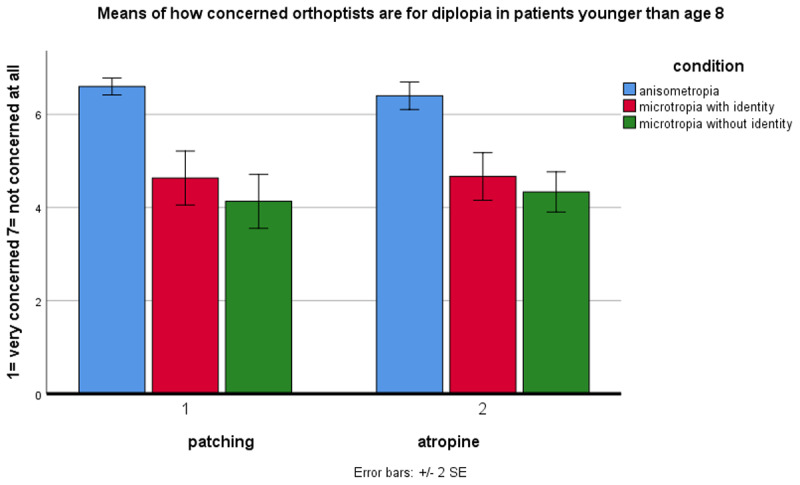
Orthoptists’ concern about diplopia during amblyopia treatment in patients younger than age 8.

**Figure 2:**
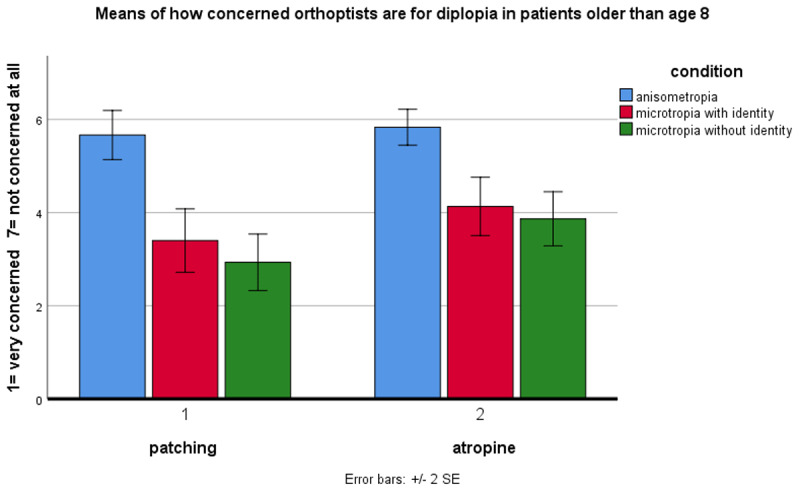
Orthoptists’ concern about diplopia during amblyopia treatment in patients older than age 8.

Orthoptists were somewhat concerned about diplopia when treating amblyopia in younger (<8 years) (Figure 1) and older (>8 years) patients (Figure 2). A 3-factor repeated measures ANOVA showed a significant main effect of condition (p < 0.001). Orthoptists had greater concern about diplopia in microtropia with identity (m = 4.208) and microtropia without identity (m = 3.817) than anisometropia (m = 6.125). Post-hoc comparisons (with Bonferroni adjustment) showed significantly greater concern about diplopia in microtropia without identity compared to with identity (p < 0.027) and to anisometropia (p < 0.001), and in microtropia with identity compared to anisometropia (p < 0.001). Overall, there was a significant main effect of age (p < 0.001) with greater concern about diplopia when treating amblyopia in older patients (m = 4.306) than younger patients (m = 5.128). Overall, concern about diplopia was not significantly different when patching (m = 4.561) compared to atropine (m = 4.872) (p > 0.05).

However, there was a significant two-way interaction (age × treatment, p < 0.004) showing greater concern about diplopia in older patients when patching (m = 4.0) compared to using atropine (m = 4.61). When treating amblyopia in microtropia, there was significantly greater concern for diplopia when patching compared to atropine (two-way interaction, condition × treatment, p < 0.006). There were no significant interactions between condition × age (p > 0.05), and condition × age × treatment (p > 0.05).

### Atropine vs patching

Orthoptists reported that there was no significant difference in risk of diplopia between atropine and patching (p > 0.05). However, comments highlighted a preference for patching (n = 3), a lack of experience using atropine (n = 6) and a view that atropine was not suitable in microtropia due to VA being too poor (n = 1).

### Preventing diplopia

In microtropia, to prevent diplopia, orthoptists reported it was most important to ask the parents to monitor diplopia at home, followed by monitoring sensory fusion, monitoring the angle of deviation and to stop occlusion before the patient reaches equal VA (Table 1). For these four methods considered most important to ‘prevent diplopia’, it was significantly more important to do this in microtropia with and without identity than anisometropia (p < 0.05). The difference between microtropia with and without identity was not significant (p > 0.05) (one-factor ANOVA with Bonferroni correction for six comparisons). Giving orthoptic exercises and monitoring the prism fusion range were reported to be the least important to prevent diplopia. Three orthoptists also commented in free text that their decision depended on the patient’s binocularity.

**Table 1:** Results showing how important orthoptists considered different measures to prevent diplopia. 1 = very important 7 = not important at all.


**Asking the parents to monitor whether diplopia is reported at home**	Mean

Anisometropia	3.1

Anisometropia with microtropia with identity	2.3

Anisometropia with microtropia without identity	2.1

**Monitor sensory fusion and/or stereopsis (Worth lights, TNO etc)**	Mean

Anisometropia	3.2

Anisometropia with microtropia with identity	2.6

Anisometropia with microtropia without identity	2.5

**Monitor the angle of deviation**	Mean

Anisometropia	4.7

Anisometropia with microtropia with identity	3.3

Anisometropia with microtropia without identity	2.9

**Stop occlusion or atropine treatment before they reach equal visual acuity**	Mean

Anisometropia	6

Anisometropia with microtropia with identity	3.4

Anisometropia with microtropia without identity	3.3

**Monitor convergent and divergent fusion range (using a prism bar)**	Mean

Anisometropia	4.8

Anisometropia with microtropia with identity	4.1

Anisometropia with microtropia without identity	4.4

**Give orthoptic exercises**	Mean

Anisometropia	6

Anisometropia with microtropia with identity	6.2

Anisometropia with microtropia without identity	6.1


### Informing parents about the risk of diplopia

Orthoptists were significantly more likely to inform patients or parents about the risk of diplopia (1 = never–7 = always) in microtropia with identity (m = 5.0) and microtropia without identity (m = 5.1) than anisometropia (m = 3.0) (p < 0.001). Older patients (m = 5.1) were significantly more likely to be informed about the risk of diplopia than younger patients (m = 3.7) (p < 0.001).

### Action to take if suspecting diplopia

Orthoptists reported they would take several different actions if diplopia was suspected in a patient. There were slight but not significantly different actions in the different conditions. Actions included stopping amblyopia treatment (n = 21–22), seeing patients more frequently (n = 21–23), asking the parent to monitor diplopia at home (n = 16–17), reducing amblyopia treatment (n = 11–13), swapping from patching to atropine (n = 1–2), or swapping from atropine to patching (n = 1). None reported they would continue treatment unchanged. Some orthoptists also gave additional comments explaining they also would monitor suppression depth (n = 1), perform new cycloplegic refraction (n = 1), and prescribe reverse occlusion (n = 1).

### When to stop amblyopia treatment

**Figure 3:**
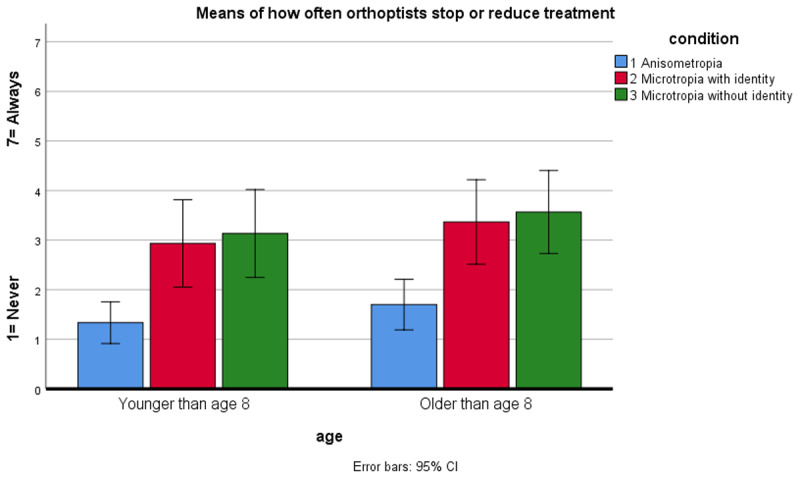
Mean results of how often orthoptists would stop or reduce amblyopia treatment in a patient who had not complained about diplopia and VA was still improving.

Most orthoptists reported they would monitor a patient undergoing amblyopia treatment every 10–12 weeks in anisometropia and every 7–9 or 10–12 weeks in microtropia (with and without identity) for both younger and older patients. If they suspected diplopia in a patient undergoing amblyopia treatment, most orthoptists would stop or reduce treatment, ask parents to monitor at home and see the patient more frequently. The presence of microtropia did not make any difference to these actions. However, results showed that orthoptists sometimes stopped treatment even if the patient had not complained about diplopia and VA was still improving (1 = never–7 = always) (Figure 3). There was no significant difference in the responses for younger and older patients (p > 0.05). In both age groups, orthoptists reported they would stop treatment in microtropia with identity (younger m = 2.9, older m = 3.4) and microtropia without identity (younger m = 3.1, older m = 3.36) significantly more often than in anisometropia (younger m = 1.3, older m = 1.7) (p < 0.001).

### VA outcomes

**Figure 4:**
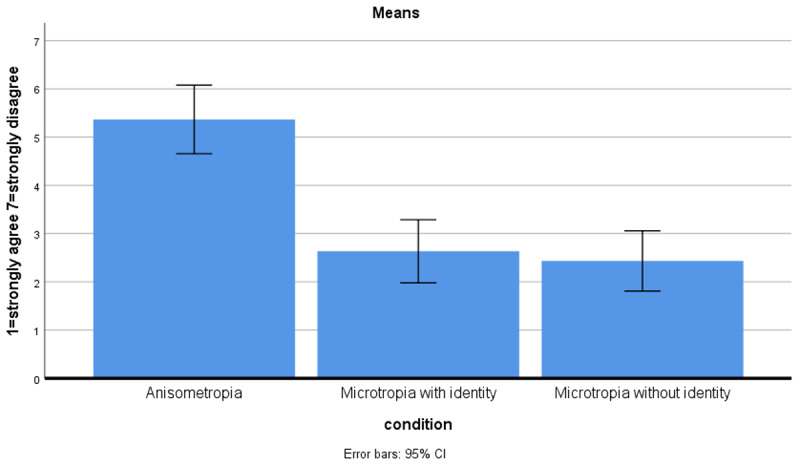
How much orthoptists agreed equal VA was difficult to achieve in patients with amblyopia.

Figure 4 shows how much the respondents agreed that equal VA was difficult to achieve in the three different conditions (1 = strongly agree–7 = strongly disagree). Orthoptists reported that equal VA was significantly more difficult to achieve in microtropia with identity (m = 2.6) and microtropia without identity (m = 2.4) compared to anisometropia (m = 5.4) (p < 0.001). There was no significant difference in their opinions on how difficult it was to achieve equal VA in microtropia with and without identity (p > 0.05).

Most orthoptists (n = 22) expected equal VA in anisometropia at the end of amblyopia treatment, or 1–2 lines worse than the fellow eye (n = 6). In microtropia with and without identity, most (n = 22) reported VA of 1–2 lines worse than the fellow eye or 3–4 lines (n = 6) to be a good final outcome.

## Discussion

A questionnaire was sent to orthoptists working in Scandinavia to explore their opinions of amblyopia treatment in microtropia. Responses to questions about amblyopia treatment in microtropia, both with and without identity, were compared to amblyopia treatment in anisometropia only to try and understand the influence of microtropia on clinical decision-making. Orthoptists reported when treating amblyopia, the presence of microtropia raised more concern about diplopia and led to being more likely to stop amblyopia treatment, even if VA was improving and there was no diplopia. Equal VA was considered more difficult to achieve in microtropia, and orthoptists reported it was acceptable to have residual amblyopia in microtropia compared to anisometropia.

### Concern about diplopia

The reported concerns about diplopia when treating amblyopia in microtropia contrasts with the more recent evidence that diplopia is extremely rare in these circumstances. A questionnaire study estimated there were 24 cases of intractable diplopia following amblyopia treatment over a five-year period in the UK (Newsham and O’Connor, 2016). A later one-year prospective UK observational study reported no cases of intractable diplopia following amblyopia treatment (Newsham, O’Connor and Harrad, 2018). PEDIG (2005) reported only four cases of transient diplopia in 507 patients undergoing two to six hours of patching for amblyopia. The two studies reporting diplopia following amblyopia treatment did not comment on whether microtropia was present or not (Newsham and O’Connor, 2016; PEDIG, 2005). Recent studies of amblyopia treatment in microtropia have not reported any incidences of diplopia (Migliorini *et al*., 2019; Lysons and Tapley, 2018). Diplopia following amblyopia treatment appears to be more frequently reported in older orthoptic literature (Lang, 1974) and specifically in older children with abnormal correspondence (Campos, 1995; von Noorden, 1996: 344). Older literature reported that bifoveal fixation could not be achieved (Bagolini and Campos, 1983) and cautioned against ‘too much’ amblyopia treatment (von Noorden, 1996, p. 344). However, there is little evidence reported specifically in microtropia.

Orthoptists in this study often informed families of the risk of diplopia when treating amblyopia and more often to those with microtropia than anisometropia. Families of older patients were informed significantly more often than younger. Whilst information about diplopia allows the orthoptist to intervene in order to alleviate the symptoms (Hoole and Barrow, 2017), diplopia after amblyopia treatment has not been reported more frequently in older patients (PEDIG, 2005). Orthoptists were also more likely to stop amblyopia treatment in the presence of a microtropia, even if there was no sign of diplopia and VA was improving, regardless of patient age. The questionnaire did not explore reasons for greater concern about diplopia in microtropia compared to anisometropia, but three orthoptists commented that the risk of diplopia depended on the patient’s BSV. Due to the study design, more detailed information about BSV and microtropia type could not be explored further; however, it is acknowledged that diplopia is a known risk in anisometropia due to aniseikonia and poorer fusion abilities (South *et al*., 2019). It has also been suggested that greater amounts of anisometropia are more likely to have a co-existing microtropia with identity (Weakley, 2001), and microtropia without identity may be regarded as less stable due to fixation in the amblyopic eye (Tomac, Sener and Sanac, 2002) or reduced fusion (Tychsen, 2005).

### Preventing diplopia

To prevent diplopia when treating amblyopia in microtropia, orthoptists reported it was important to monitor diplopia at home, sensory fusion and the angle of deviation, as well as stop occlusion before equal VA was reached. Fusion loss or increase in the angle of deviation have been reported as signs of decompensation in MFS and may cause diplopia (Ing *et al*., 2014). Other reported risk factors of decompensation in MFS include undercorrection of hypermetropia (Lang, 1974), a vertical deviation and poor horizontal fusional amplitudes (Siatkowsky, 2011). Before answering the questions, the respondents were informed that they should consider the patient to be wearing glasses (full correction) following a recent refraction under cycloplegia; therefore, it is not possible to discuss their opinions of undercorrection of hypermetropia on diplopia. Siatkowsky (2011) did not find that a presence of amblyopia, history of patching, or former orthoptic exercises were found more frequently in patients who had decompensated MFS. Hunt and Keech (2005) suggested that in decompensated MFS, a history of amblyopia would make a new adaptation to suppression easier, but residual amblyopia was not necessary for suppression. Therefore, limiting amblyopia treatment in patients with MFS was not recommended (Siatkowski, 2011; Hunt and Keech, 2005).

The orthoptists in this study did not consider monitoring fusion range and giving orthoptic exercises important to prevent diplopia. In contrast, Siatkowsky (2011) reported that fusional amplitudes in MFS often diminish with time and suggested measuring fusional amplitudes and giving orthoptic exercises to patients with reduced fusional amplitudes.

Four orthoptists commented that it was important to monitor depth of suppression with a filter bar even though the patients had microtropia (and therefore BSV). Microtropia was defined in the introduction of the questionnaire, but it may have been unclear to some respondents that these patients had BSV and demonstrated fusion (and not suppression). A similar finding was observed when UK orthoptists were asked about assessing the density of suppression, as 18% reported they would measure this in patients with fusion (Newsham and O’Connor, 2016).

Not all patients with microtropia may be correctly diagnosed and differentiated from pure anisometropia. A visuoscope is needed to assess monocular fixation and can therefore diagnose a microtropia with identity (Garretty, 2021). A 4 PD prism reflex test can only confirm a foveal suppression scotoma, which could be due to reduced vision or reduced stereopsis caused by anisometropia (Rutstein, 2018). More orthoptists used the 4 PD prism reflex test than the visuoscope to diagnose microtropia, which also could be a factor when making decisions for treatment.

### Patching vs atropine

Orthoptists did not report that patching or atropine gave greater concern about the risk of diplopia in younger patients. However, in patients older than age eight, greater concern about diplopia was reported in patients with microtropia when patching. This was an unexpected result, as several orthoptists commented they preferred patching over atropine. It is possible that more orthoptists responded ‘neutral’ because of a lack of experience with atropine, while they responded slightly concerned when using patching, giving the mean a higher score towards being more concerned when patching. It could also mean that they were truly less concerned when using atropine because of the preservation of sensory fusion compared to a complete occlusion with a patch. This result is not in agreement with UK orthoptists, who reported being restrictive in using atropine for older children in cases of systemic side effects or diplopia (Newsham and Connor, 2016). However, it is acknowledged that UK practice may have changed with more recent experiences with atropine and patching.

### VA outcomes

Orthoptists in this study reported equal VA was significantly more difficult to achieve in microtropia with and without identity compared to pure anisometropia. Cotter *et al*. (2006) reported that amblyopia resolved with glasses alone in one third of anisometropia cases. However, they did not exclude microtropia with identity from the patient group, so it is not clear whether patients with microtropia achieved equal VA as often as those with bifoveal BSV or if they belonged to the 2/3 who needed patching in addition to glasses. In microtropia, orthoptists in this study expected no better than a one- to two-line difference between eyes after treatment. This is consistent with the findings of Migliorini (2019) and Lysons and Tapley (2018). Lysons and Tapley (2018) reported equal VA in pure anisometropia after treatment but a small interocular difference in VA in microtropia. Migliorini (2019) reported VA did not improve beyond an average of 0.1 LogMAR in the microtropic eye. In contrast to these findings, Houston *et al*. (1998) reported that 13 of 30 patients with microtropia achieved an equal VA of –0.1 LogMAR and that 9 of 30 patients no longer had microtropia.

In general, orthoptists did not stop or reduce amblyopia treatment more often in patients older than age 8 compared to younger, consistent with findings that amblyopia treatment is effective also in older children with both strabismic and anisometropic amblyopia (PEDIG, 2005) and in patients with microtropia (Migliorini, 2019).

It is worth noting that microtropic patients who received less patching (1–4 hours daily) reported poorer VA outcomes (Everhard-Halm and Wenniger-Prick, 1989) than those who received more patching (up to 8 hours daily) (Houston *et al*., 1998; Migliorini *et al*., 2019). This contrasts with the results from PEDIG (2005) reporting the same improvement in VA with 2 hours of patching, compared to 6 hours, in moderate amblyopia. However, more than 6 hours of patching was not specifically studied. A recent study by Proudlock *et al*. (2024) reported better VA outcomes with an intensive patching regime, 10 hours daily, 6 days a week, after only a short period of glasses use, compared to those who did not start patching until 18 weeks after glasses alone. However, few studies have specifically reported amblyopia treatment outcomes in microtropia, and this remains an area for further study.

### Differences in orthoptists working in different countries

Scandinavian orthoptists have commonly either trained in Sweden or other European countries such as Germany, Switzerland, Portugal, Italy or the UK. Recently, a European Diploma for Orthoptists (EDORTH) was developed as there are differences in the various European training programs for orthoptists. However, analysis of the results from this survey by country of training was beyond the scope of this questionnaire. The amount of orthoptic experience of the respondents was mean 19.5 years. The new training program that was mapped on EDORTH commenced in 2021, meaning that the orthoptists who graduated from this course would not have been a part of the survey. It is therefore not possible to know if recently educated orthoptists would recommend different amblyopia treatment in microtropia than colleagues educated many years ago. The small sample size also prohibited comparison of results by age of respondent in this study.

### Limitations of the study and ideas for future research

A questionnaire can limit the ability to explore the respondent’s understanding of the questions and their opinions in more detail. Although additional comment boxes with free text were included, some of the questions may have been difficult to answer without additional information or clinical context. Expectation bias could occur; for example, respondents may have had an expectation that microtropia without identity should raise more concern as this condition was listed last. The response rate was only 17% of all members of the SOA (n = 170), and therefore responses may not have been representative of all Scandinavian orthoptists. This questionnaire was anonymous, which allowed respondents to be completely honest. However, to gain more specific information about clinical decision-making in microtropia, future research could include interviews or focus groups to qualitatively explore the influence of different clinical factors in decision-making. The impact of newer education programs for orthoptists, such as EDORTH, on clinical decision-making could also be investigated.

A prospective study of amblyopia treatment specifically in microtropia may also provide information about VA outcomes, fixation pattern, diplopia and BSV during treatment and could potentially investigate decompensation.

## Conclusion

In this study, during amblyopia treatment, orthoptists working in Scandinavia considered patients with microtropia to be more at risk of diplopia than patients with anisometropia only. Microtropia without identity was perceived to be at greater risk of diplopia than microtropia with identity. Orthoptists were more likely to stop amblyopia treatment in microtropia even if VA was improving and no diplopia was present. In microtropia, equal VA was thought to be more difficult to achieve, and a small amount of residual amblyopia was expected. There was no perceived difference in diplopia risk between patching and atropine. These findings contrast with the more recent evidence reporting diplopia is extremely rare in microtropia and following amblyopia treatment. The precise reasons why orthoptists in this study reported treating amblyopia differently in patients with microtropia compared to anisometropia are not clear, but the influence of older literature has been discussed as a possible factor.

## Additional File

The additional file for this article can be found as follows:

10.22599/bioj.316.s1 Supplementary File 1. Appendix. Questionnaire.

## References

[1] Ansons, A.M. and Davis, H. (2014) Diagnosis and management of ocular motility disorders [electronic resource]. Fourth edition. Chichester, West Sussex; Hoboken, New Jersey: Wiley. Available at: 10.1002/9781118712368

[2] Bagolini, B. and Campos, E.C. (1983) ‘Practical usefulness of anomalous binocular vision for the strabismic patient’, International Ophthalmology, 6(1), pp. 19–26. Available at: 10.1007/BF00137369 6826288

[3] Campos, E. (1995) ‘Amblyopia’, Survey of Ophthalmology, 40(1), pp. 23–39. Available at: 10.1016/S0039-6257(95)80044-1 8545799

[4] Cotter, S.A. *et al*. (2006) ‘Treatment of anisometropic amblyopia in children with refractive correction’, Ophthalmology, 113(6), pp. 895–903. Available at: 10.1016/j.ophtha.2006.01.068 16751032 PMC1790727

[5] Everhard-Halm, Y.S. and Maillette de Buy Wenniger-Prick, L.J.J.M. (1989) ‘Amblyopia treatment in microstrabismus’, British Orthoptic Journal, 46, pp. 109–111.

[6] Garretty, T. (2021) ‘The agreement between the Irvine 4 dioptre prism test and assessment of ocular fixation in microtropia with identity’, Strabismus, 29(2), pp. 81–85. Available at: 10.1080/09273972.2021.1914675 33886409

[7] Hoole, J. and Barrow, N. (2017) ‘Diplopia following short treatment for moderate amblyopia’, Strabismus, 25(3), pp. 166–170. Available at: 10.1080/09273972.2017.1350725 28937884

[8] Houston, C.A. *et al*. (1998) ‘Clinical characteristics of microtropia—is microtropia a fixed phenomenon?’, British Journal of Ophthalmology, 82(3), pp. 219–330. Available at: 10.1136/bjo.82.3.219 9602615 PMC1722512

[9] Hunt, M.G. and Keech, R.V. (2005) ‘Characteristics and course of patients with deteriorated monofixation syndrome’, Journal of AAPOS, 9(6), pp. 533–536. Available at: 10.1016/j.jaapos.2005.08.004 16414518

[10] Ing, M.R. *et al*. (2014) ‘The stability of the monofixation syndrome’, American Journal of Ophthalmology, 157(1), pp. 248–253. Available at: 10.1016/j.ajo.2013.08.024 24200233

[11] Jampolsky, A. (1951) ‘Retinal correspondence in patients with small degree strabismus’, A.M.A. Archives of Ophthalmology, 45(1), pp. 18–26. Available at: 10.1001/archopht.1951.01700010021002 14789286

[12] Lang, J. (1974) ‘Management of microtropia’, British Journal of Ophthalmology, 58(3), pp. 281–292. Available at: 10.1136/bjo.58.3.281 4834601 PMC1214740

[13] Lang, J. (1983) ‘Microtropia’, International Ophthalmology, 6(1), pp. 33–36. Available at: 10.1007/BF00137371. 6826290

[14] Lysons, D. and Tapley, J. (2018) ‘Is microtropia a reliable indicator of the presence of amblyopia in anisometropic patients?’, Strabismus, 26(3), pp. 118–121. Available at: 10.1080/09273972.2018.1503308 30060696

[15] Matsuo, T. *et al*. (2003) ‘Long-term visual outcome in primary microtropia’, Japanese Journal of Ophthalmology, 47(5), pp. 507–511. Available at: 10.1016/S0021-5155(03)00105-9 12967869

[16] Migliorini, R. *et al*. (2019) ‘Anisometropia and isoametropia associated with microstrabismus: Effectiveness of late anti-amblyopia treatment’, La Clinica Terapeutica, 170(5), pp. e339–44. Available at: 10.7417/CT.2019.2158. 31612190

[17] Newsham, D. *et al*. (2018) ‘Incidence, risk factors and management of intractable diplopia’, British Journal of Ophthalmology, 102(3), pp. 393–397. Available at: 10.1136/bjophthalmol-2017-310454 28659388

[18] Newsham, D. and O’Connor, A.R. (2016) ‘Assessment of the density of suppression to identify risk of intractable diplopia in the United Kingdom’, Strabismus, 24(2), pp. 45–50. Available at: 10.3109/09273972.2016.1170048 27220683

[19] Parks, M. (1969) ‘Th monofixation syndrome’, Transactions of the American Ophthalmological Society, 67, pp. 609–657. 5381308 PMC1310353

[20] Pediatric Eye Dis Investigators Gr and Pediatric Eye Disease Investigator Group (2005) ‘Randomized Trial of Treatment of Amblyopia in Children Aged 7 to 17 Years’, Archives of ophthalmology (1960), 123(4), pp. 437–447. Available at: 10.1001/archopht.123.4.437 15824215

[21] Proudlock, F.A. *et al*. (2024) ‘Extended optical treatment versus early patching with an intensive patching regimen in children with amblyopia in Europe (EuPatch): A multicentre, randomised controlled trial’, The Lancet, 403(10438), pp. 1766–1778. Available at: 10.1016/S-0140-6736(23)02893-3 38704172

[22] Rowe, F.J. (2012) Clinical Orthoptics. 3rd ed. Chichester: Wiley-Blackwell. Available at: 10.1002/9781118702871

[23] Rutstein, R.P. (2018) ‘Letter to editor: “is microtropia a reliable indicator for the presence of amblyopia in anisometropic patients?” by Lysons and Tapley’, Strabismus, 26(4), pp. 210–210. Available at: 10.1080/09273972.2018.1524916 30285526

[24] Siatkowski, M.R. (2011) ‘The decompensated monofixation syndrome’, Transactions of the American Ophthalmological Society, 109, pp. 232–250. 22253490 PMC3259678

[25] South, J. *et al*. (2019) ‘Aniseikonia and anisometropia: Implications for suppression and amblyopia’, Clinical and Experimental Optometry, 102(6), pp. 556–565. Available at: 10.1111/cxo.12881 30791133

[26] Tomaç, S. *et al*. (2002) ‘Clinical and sensorial characteristics of microtropia’, Japanese Journal of Ophthalmology, 46(1), pp. 52–58. Available at: 10.1016/S0021-5155(01)00470-1 11853714

[27] Tychsen, L. (2005) ‘Can ophthalmologists repair the brain in infantile esotropia? Early surgery, stereopsis, monofixation syndrome, and the legacy of Marshall Parks’, Journal of AAPOS, 9(6), pp. 510–521. Available at: 10.1016/j.jaapos.2005.06.007 16414515

[28] Von Noorden, G.K. (1996) Binocular vision and ocular motility: Theory and management of strabismus. 5th ed. Mosby.

[29] Weakley, D.R. (2001) ‘The association between nonstrabismic anisometropia, amblyopia, and subnormal binocularity’, Ophthalmology (Rochester, Minn.), 108(1), pp. 163–171. Available at: 10.1016/S0161-6420(00)00425-5 11150283

