# Supplementary material for: Opinions on Amblyopia Treatment in Microtropia – A Questionnaire Study of Orthoptists in Scandinavia
Source: Br Ir Orthopt J. 2024 Nov 13;20(1):226–34. doi: 10.22599/bioj.316 (PMC11568811; doi:10.22599/bioj.316)
Supplement: Supplementary File 1. — Appendix. Questionnaire. [file bioj-20-1-316-s1.pdf]

1 Appendices

1.1 Appendix A presents the ethics approval letter granted by the University ethics committee. The questionnaire form in Appendix B consists of a participant information sheet and consent form introductory to the 27 questions which the participants received as a Google Form online. Histograms of response frequencies will be displayed in Appendix C to show the distribution and why it was considered mainly normal. This was important for the choice of statistical analysis. The statistical calculations are presented in Appendix D. Some of the histograms in Appendix C were considered not normal, and non-parametric analysis were performed for these questions in addition to the parametric analysis. The results are presented in Appendix E. The sample size was calculated with the software "G*Power" as shown in Appendix F. When the results from the respondents were investigated, one outlier stood out, and this is explained in Appendix G. At last, a table was put up to see if any differences could be found between countries on chosen questions. The results are reported in the results section (section 3.4.5), but additional results are shown in Appendix H.

1.2 Appendix A. Approval letter

1.3 Appendix B. Questionnaire with Participant information sheet and Consent form

1.4 Appendix C. Histograms of response frequencies

1.5 Appendix D. Statistical analysis

1.6 Appendix E. Description of non-parametric analysis on q.18,19 and 20

1.7 Appendix F. Sample size

1.8 Appendix G. Removal of outlier tables

1.9 Appendix H. Examples of differences in means between countries

Appendix A Approval letter

Appendix B. Questionnaire

The University Of Sheffield.

Invitation to participate in a questionnaire-based study

"Orthoptists' views on amblyopia treatment in anisometropia with microtropia"

Participant information (version1)

You are being invited to take part in a University of Sheffield research study by completing a questionnaire. Before you decide whether or not to participate, it is important for you to understand why the research is being done, and what it will involve. Please take time to read the following information carefully. You can contact us if there is anything that is not clear or if you would like more information.

The purpose of this research is to evaluate orthoptists' views on amblyopia treatment in anisometropia with and without co-existing microtropia. This research is being undertaken as part of a Masters research project. The data collected from this questionnaire will be used in a Masters dissertation and to consider if further research on the topic is recommended. We plan to publish the findings from this research in a medical journal.

You have been invited to complete the questionnaire because you are a member of the Scandinavian Orthoptic Association (SOA). We are looking to gather the views of all currently working orthoptists in Scandinavia with a membership in SOA. The questionnaire should take approximately 30 minutes to complete, and your responses will be anonymous.

It is up to you to decide whether or not to take part in this research study. If you do decide to take part, you can proceed to the next section of this questionnaire where you will be asked to give your consent. The information and questionnaire will only be given in English, so it is important that you understand the information before you give your consent. If you choose to give your consent you will proceed to the questions within the questionnaire.

Your responses will not be recorded until you click "submit" at the end of the questionnaire. That means you can withdraw from the research at any time before that without any negative consequences. You do not have to give a reason. When you do submit your responses, they will be anonymous, and the researcher will not be able to recognise your identity. We will gather and register the information provided by you, but we cannot trace your answers back to you. Your contribution will be assigned a number/code that distinguishes you from the others.

You have six weeks to answer the questionnaire. After that, the form will no longer be available. An e-mail will be sent two weeks before the deadline as a reminder to complete the questionnaire, if you have not already done so.

The information gathered from the questionnaire will be securely stored for ten years in line with University of Sheffields' standard practice.

This project is not sponsored and does not receive any form of financial support.

We are very grateful for your response as it will give a valuable contribution to the interpretation of the results. We also thank you for the time and effort you spend filling in the questionnaire.

If you have concerns about safeguarding relating to this project, you can contact any of the people listed below to raise your concerns anonymously or with your identity known.

Project supervisor and Designated Safeguarding Contact:

Gemma Arblaster g.arblaster@sheffield.ac.uk

Contacts independent of the research team:

Head of Head Sciences School: Prof Tracey Moore tracey.moore@sheffield.ac.uk Tel (+44) 114 222 2056

Research Ethics and Integrity Manager, Research Services, University of Sheffield: Lindsay Unwin l.v.unwin@sheffield.ac.uk Tel (+44) 114 2221443

If you have any questions about the research or the wording in the questionnaire, please contact: Investigator: Jannicke Røe, e mail: jroee1@sheffield.ac.uk

Consent

Please read the following statements and tick the final box if you agree with each statement:

I have read and understood the participant information (version 1) on the previous page.

I understand I can contact the researcher by email if I have questions about the project.

I agree to take part in the research project and understand that taking part will involve completing a questionnaire.

I understand that taking part is voluntary and that I can withdraw from the research before I click on the "submit" button at the end of the questionnaire. I do not have to give any reasons why I will not take part, and there will be no adverse consequences if I choose not to participate.

I understand my responses will not be shown to people outside the research team.

I understand and agree that my words may be quoted anonymously in publications, reports, web pages, and other research outputs.

So that the information you provide can be used legally by the researchers: Do you agree with the above statements?

Yes – tick box and click "continue" ☐

Orthoptists' views on amblyopia treatment in anisometropia with microtropia

Introduction

The purpose of this research is to evaluate orthoptists' views on amblyopia treatment in anisometropia where microtropia exists.

Questions will be asked to explore your views on amblyopia treatment in three different clinical scenarios. There are no right or wrong answers. The questionnaire aims to find out more about how microtropia may or may not influence your management decisions in amblyopia.

Please respond to each question and each clinical scenario using the scales given. You may also add 'free text comments' throughout the questionnaire if you would like to add more detail or expand on your answers.

You will not be able to save your answers, so please make sure you have time to complete and submit the questionnaire once you have started.

The three different clinical scenarios are described below:

Scenario a: anisometropia with unilateral amblyopia, no deviation and no microtropia ("pure" anisometropia).

Scenario b: anisometropia with unilateral amblyopia and microtropia *with* identity.

Scenario c: anisometropia with unilateral amblyopia and microtropia *without* identity.

For the purposes of this questionnaire the clinical scenarios are defined as the following:

Scenario a: anisometropia is defined as a difference of 1 or more dioptres (spherical equivalent) between the two eyes.

Scenario b: microtropia with identity is defined as no manifest deviation detected on cover test, parafoveal fixation and abnormal correspondence, where the angle of anomaly equals the same as the angle of eccentricity between the fovea and pseudo/parafovea.

Scenario c: microtropia without identity is defined as a small manifest deviation less than 8 dioptres detected on cover test, abnormal correspondence and central or non-absolute parafoveal fixation.

To differentiate between treatment in younger and older children, you will be asked about amblyopia treatment decisions in children younger than, or older than, 8 years of age.

When giving your views on amblyopia treatment, please assume the patient has had a recent refraction under cycloplegia and is wearing glasses with the full correction, and they are fully compliant with refractive correction, wearing the glasses full-time.

Attitudinal questions

1. How concerned are you that a patient with unilateral amblyopia, *younger than age 8*, will develop diplopia during amblyopia treatment with **patching**?

a: in “pure” anisometropia. Very concerned-not concerned at all. 1-7.

b: in anisometropia with microtropia with identity. Very concerned-not concerned at all. 1-7.

c: in anisometropia with microtropia without identity. Very concerned-not concerned at all. 1-7.

Comment: ____

2. How concerned are you that a patient with unilateral amblyopia, *older than age 8*, will develop diplopia during amblyopia treatment with **patching**?

a: in “pure” anisometropia. Very concerned-not concerned at all. 1-7.

b: in anisometropia with microtropia with identity. Very concerned-not concerned at all. 1-7.

c: in anisometropia with microtropia without identity. Very concerned-not concerned at all. 1-7.

Comment: ____

3. How concerned are you that a patient with unilateral amblyopia, *younger than age 8*, will develop diplopia during amblyopia treatment with **atropine**?

a: in “pure” anisometropia. Very concerned-not concerned at all. 1-7.

b: in anisometropia with microtropia with identity. Very concerned-not concerned at all. 1-7

c: in anisometropia with microtropia without identity. Very concerned-not concerned at all. 1-7

Comment: ____

4. How concerned are you that a patient with unilateral amblyopia, *older than age 8*, will develop diplopia during amblyopia treatment with **atropine**?

a: in “pure” anisometropia. Very concerned-not concerned at all. 1-7

b: in anisometropia with microtropia with identity. Very concerned-not concerned at all. 1-7.

c: in anisometropia with microtropia without identity. Very concerned-not concerned at all. 1-7.

Comment: ____

5. How important do you consider the following to prevent diplopia in amblyopes receiving treatment?

-monitor sensory fusion and/or stereopsis (Worth lights, TNO etc)

a: in “pure” anisometropia. Very important-not important at all. 1-7

b: in anisometropia with microtropia with identity. Very important-not important at all. 1-7

c: in anisometropia with microtropia without identity. Very important-not important at all. 1-7

Comment: ____

6. How important do you consider the following to prevent diplopia in amblyopes receiving therapy?

- Asking the parents to monitor whether diplopia is reported at home?

a: in “pure” anisometropia. Very important-not important at all. 1-7

b: in anisometropia with microtropia with identity. Very important-not important at all. 1-7

c: in anisometropia with microtropia without identity. Very important-not important at all. 1-7

Comment: ____

7. How important do you consider the following to prevent diplopia in amblyopes receiving therapy?

-Monitor the angle of deviation

a: in “pure” anisometropia. Very important-not important at all. 1-7

b: in anisometropia with microtropia with identity. Very important-not important at all. 1-7

c: in anisometropia with microtropia without identity. Very important-not important at all. 1-7

Comment: ____

8. How important do you consider the following to prevent diplopia in amblyopes receiving therapy?

-Monitor convergent and divergent fusion range (using a prism bar)

a: in “pure” anisometropia. Very important-not important at all. 1-7

b: in anisometropia with microtropia with identity. Very important-not important at all. 1-7

c: in anisometropia with microtropia without identity. Very important-not important at all. 1-7

Comment: ____

9. How important do you consider the following to prevent diplopia in amblyopes receiving therapy?

-Stop occlusion or atropine treatment before they reach equal visual acuity

a: in "pure" anisometropia. Very important-not important at all. 1-7

b: in anisometropia with microtropia with identity. Very important-not important at all. 1-7

c: in anisometropia with microtropia without identity. Very important-not important at all. 1-7

Comment: ____

10. How important do you consider the following to prevent diplopia in amblyopes receiving therapy?

-Give orthoptic exercises.

a: in "pure" anisometropia. Very important-not important at all. 1-7

b: in anisometropia with microtropia with identity. Very important-not important at all. 1-7

c: in anisometropia with microtropia without identity. Very important-not important at all. 1-7

Comment: ____

11. Please list or describe any other factors (not covered by the previous questions) you consider important to prevent diplopia during amblyopia treatment

a: in "pure" anisometropia:____

b: in anisometropia with microtropia with identity: _______________________________________

c: in anisometropia with microtropia without identity: ____________________________________

Comment: ____

12. How much do you agree or disagree with the following statement in each of the clinical scenarios? "Occlusion treatment with patching carries less risk of diplopia than atropine"

a: in "pure" anisometropia. Strongly agree-strongly disagree. 1-7

b: in anisometropia with microtropia with identity. Strongly agree-strongly disagree. 1-7

c: in anisometropia with microtropia without identity. Strongly agree-strongly disagree. 1-7

Comment: ____

13. How much do you agree or disagree with the following statement in each of the clinical scenarios? "Occlusion treatment with atropine carries less risk of diplopia than patching"

a: in "pure" anisometropia. Strongly agree-strongly disagree. 1-7

b: in anisometropia with microtropia with identity. Strongly agree-strongly disagree. 1-7

c: in anisometropia with microtropia without identity. Strongly agree-strongly disagree. 1-7

Comment: ____

14. How much do you agree or disagree with the following statement in each of the clinical scenarios? “Equal visual acuity is difficult to achieve”

a: in “pure” anisometropia. Strongly agree-strongly disagree. 1-7

b: in anisometropia with microtropia with identity. Strongly agree-strongly disagree. 1-7

c: in anisometropia with microtropia without identity. Strongly agree-strongly disagree. 1-7

Comment: ____

15. In unilateral amblyopia, what final level of visual acuity in the amblyopic eye would you typically consider to be a good outcome?

a: in “pure” anisometropia. Unsure, 5-6 lines worse than the fellow eye, 3-4 lines worse than the fellow eye, 1-2 lines worse than the fellow eye, equal, 1-2 lines better than the fellow eye

b: in anisometropia with microtropia with identity. Unsure, 5-6 lines worse than the fellow eye, 3-4 lines worse than the fellow eye, 1-2 lines worse than the fellow eye, equal, 1-2 lines better than the fellow eye

c: in anisometropia with microtropia without identity. Unsure, 5-6 lines worse than the fellow eye, 3-4 lines worse than the fellow eye, 1-2 lines worse than the fellow eye, equal, 1-2 lines better than the fellow eye

Comment: ____

Behavioral questions

16. What clinical action would you take if you suspected diplopia in a patient undergoing amblyopia treatment? (multiple answers allowed)

a: in “pure” anisometropia.

No action – continue treatment unchanged
Stop amblyopia treatment
Reduce amblyopia treatment
See patient more frequently
Ask the parent / guardian to monitor diplopia at home
Swap from patching to atropine
Swap from atropine to patching
Other: ____

b: in anisometropia with microtropia with identity

No action – continue treatment unchanged
Stop amblyopia treatment
Reduce amblyopia treatment
See patient more frequently
Ask the parent / guardian to monitor diplopia at home
Swap from patching to atropine

Swap from atropine to patching
Other: ____

c: in anisometropia with microtropia without identity

No action – continue treatment unchanged
Stop amblyopia treatment
Reduce amblyopia treatment
See patient more frequently
Ask the parent / guardian to monitor diplopia at home
Swap from patching to atropine
Swap from atropine to patching
Other: ____

17. Do you inform patients *younger than age 8* or parents about the risk of diplopia when treating for amblyopia?

a: in "pure" anisometropia. Never-always. 1-7

b: in anisometropia with microtropia with identity. Never-always. 1-7

c: in anisometropia with microtropia without identity. Never-always. 1-7

Comment: ____

18. Do you inform patients *older than age 8* or parents about the risk of diplopia when treating for amblyopia?

a: in "pure" anisometropia. Never-always. 1-7

b: in anisometropia with microtropia with identity. Never-always. 1-7

c: in anisometropia with microtropia without identity. Never-always. 1-7

Comment: ____

19. Would you stop or reduce treatment for amblyopia before equal visual acuity is achieved in patients *younger than age 8* even if the patient has not complained about diplopia and vision is still improving?

a: in "pure" anisometropia. Never-always. 1-7

b: in anisometropia with microtropia with identity. Never-always. 1-7

c: in anisometropia with microtropia without identity. Never-always. 1-7

Comment: ____

20. Would you stop or reduce treatment for amblyopia before equal visual acuity is achieved in patients *older than age 8* even if the patient has not complained about diplopia and vision is still improving?

a: in “pure” anisometropia. Never-always. 1-7

b: in anisometropia with microtropia with identity. Never-always. 1-7

c: in anisometropia with microtropia without identity. Never-always. 1-7

Comment: ____

21. How often would you like to see a patient undergoing amblyopia treatment *younger than age 8?*

a: in “pure” anisometropia. Every 1-3 weeks, 4-6 weeks, 7-9 weeks, 10-12 weeks, 13-17 weeks, 18-21 weeks, 22-24 weeks

b: in anisometropia with microtropia with identity. Every 1-3 weeks, 4-6 weeks, 7-9 weeks, 10-12 weeks, 13-17 weeks, 18-21 weeks, 22-24 weeks

c: in anisometropia with microtropia without identity. Every 1-3 weeks, 4-6 weeks, 7-9 weeks, 10-12 weeks, 13-17 weeks, 18-21 weeks, 22-24 weeks

Comment: ____

22. How often would you like to see a patient undergoing amblyopia therapy *older than age 8?*

a: in “pure” anisometropia. Every 1-3 weeks, 4-6 weeks, 7-9 weeks, 10-12 weeks, 13-17 weeks, 18-21 weeks, 22-24 weeks

b: in anisometropia with microtropia with identity. Every 1-3 weeks, 4-6 weeks, 7-9 weeks, 10-12 weeks, 13-17 weeks, 18-21 weeks, 22-24 weeks

c: in anisometropia with microtropia without identity. Every 1-3 weeks, 4-6 weeks, 7-9 weeks, 10-12 weeks, 13-17 weeks, 18-21 weeks, 22-24 weeks

Comment: ____

23. What would be your preferred management plan *for a 5-year-old* who has been receiving daily 6-hour patching treatment for amblyopia. The visual acuity has been stable at 20/25 for two visits despite compliant patching treatment.

a: in “pure” anisometropia. Continue patching, stop patching, reduce patching, increase patching, Swap from patching to atropine

b: in anisometropia with microtropia with identity. Continue patching, stop patching, reduce patching, increase patching, Swap from patching to atropine

c: in anisometropia with microtropia without identity. Continue patching, stop patching, reduce patching, increase patching, Swap from patching to atropine

Comment: ____

24. What would be your preferred management plan *for a 10-year-old* who has been receiving daily 6-hour patching treatment for amblyopia. The visual acuity has been stable at 20/25 for two visits despite compliant patching treatment

a: in “pure” anisometropia. Continue patching, stop patching, reduce patching, increase patching, Swap from patching to atropine

b: in anisometropia with microtropia with identity. Continue patching, stop patching, reduce patching, increase patching, Swap from patching to atropine

c: in anisometropia with microtropia without identity. Continue patching, stop patching, reduce patching, increase patching, Swap from patching to atropine

Comment: ____

25. Which tests do you perform to diagnose a microtropia? (multiple answers allowed)

-Visual acuity

-Cover test

-Sensory fusion tests

-Motor fusion tests

-Stereotests

-4 dioptre prism reflex test

-Fixation with a visuoscope

-Measurement of refractive error

-I wouldn’t diagnose a microtropia

-Other: ____

26. How many years of experience do you have as an orthoptist? ________years

27. Which country do you practice in? (voluntary answer) ______________________

Additional comments (optional): ____

____

Appendix C

Histograms of responses

Question 1

See section 2.1

Question 2

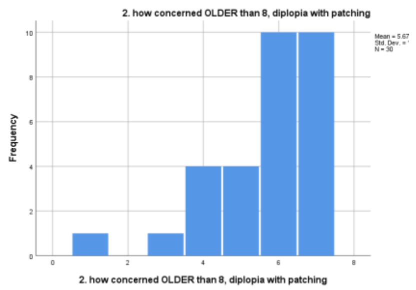

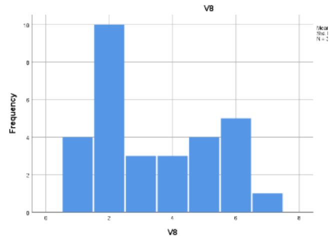

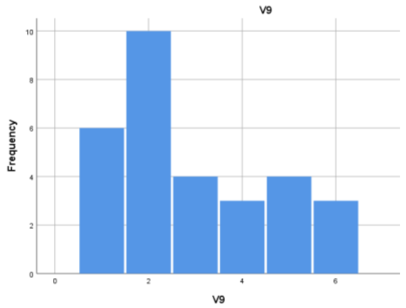

Anisometropia **microtropia with identity** **microtropia without identity**

Question 3

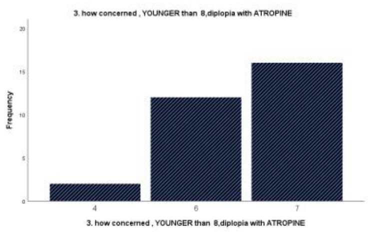

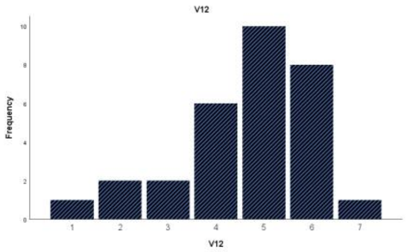

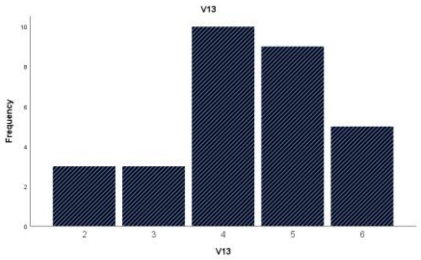

Anisometropia **microtropia with identity** **microtropia without identity**

Question 4

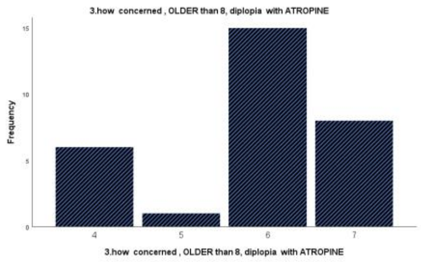

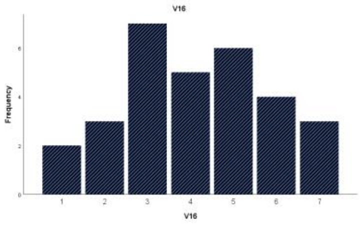

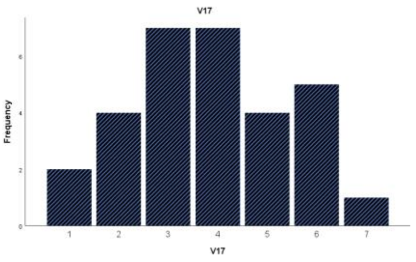

Anisometropia **microtropia with identity** **microtropia without identity**

Question 5

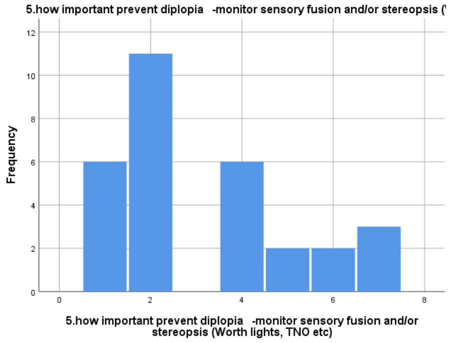

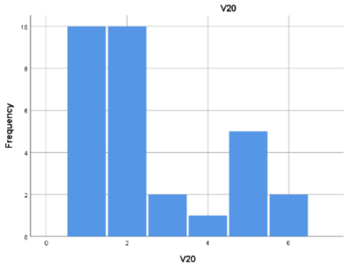

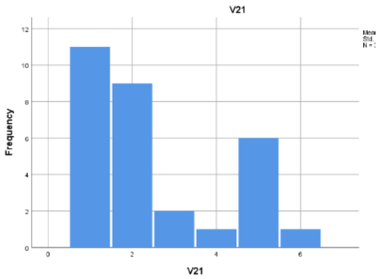

Anisometropia **microtropia with identity** **microtropia without identity**

Question 6

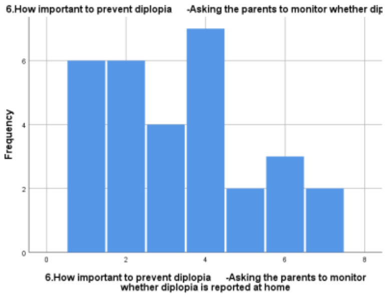

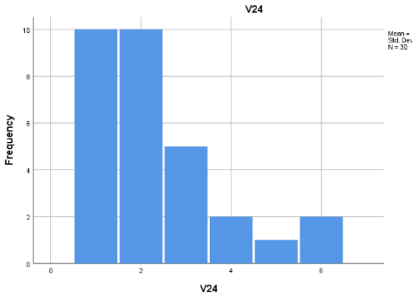

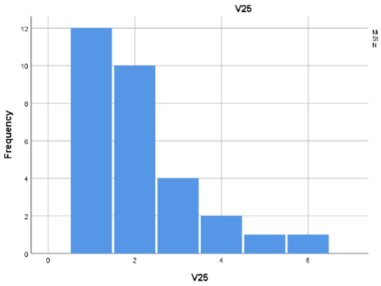

Anisometropia **microtropia with identity** **microtropia without identity**

Question 7

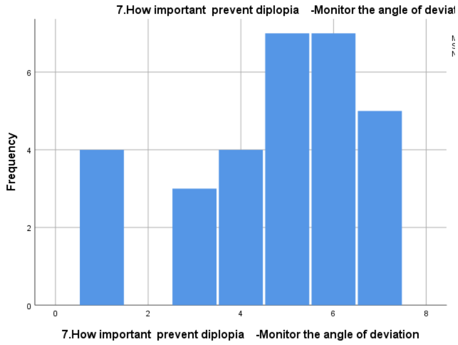

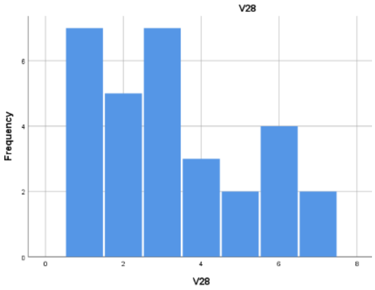

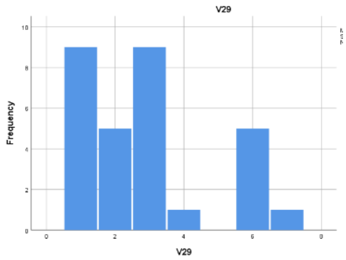

Anisometropia **microtropia with identity** **microtropia without identity**

Question 8

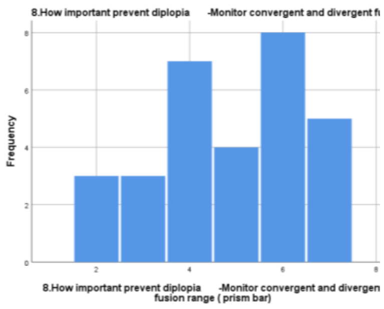

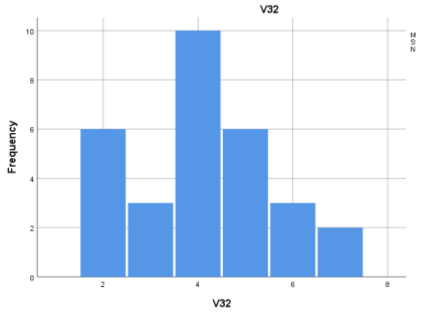

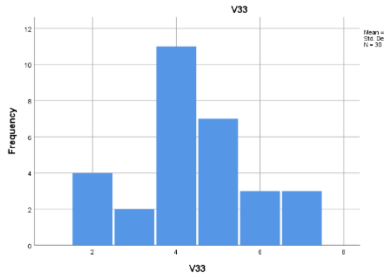

Anisometropia **microtropia with identity** **microtropia without identity**

Question 9

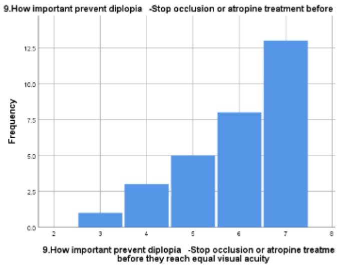

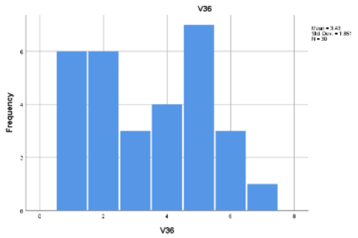

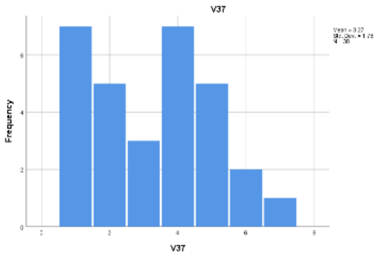

Anisometropia **microtropia with identity** **microtropia without identity**

Question 10

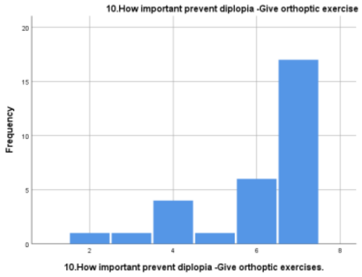

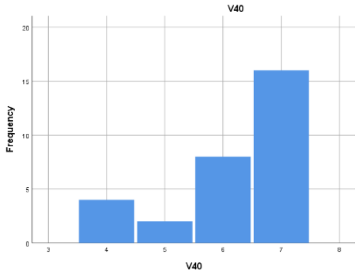

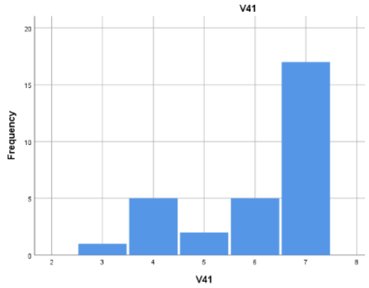

Anisometropia **microtropia with identity** **microtropia without identity**

Question 12

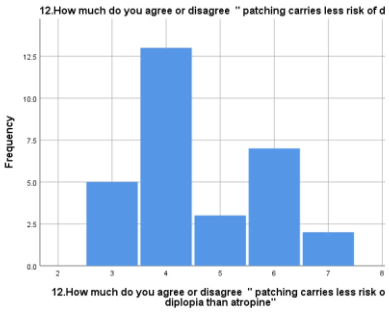

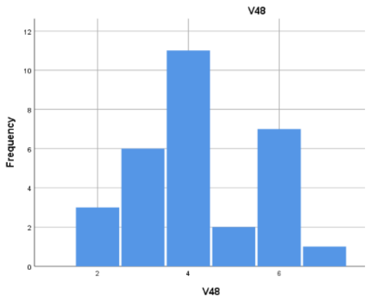

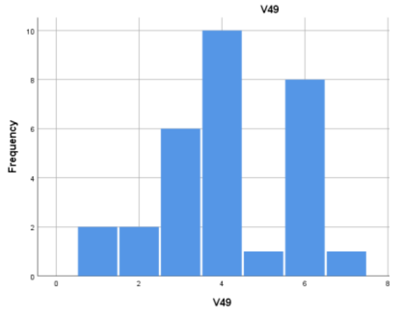

Anisometropia **microtropia with identity** **microtropia without identity**

Question 13

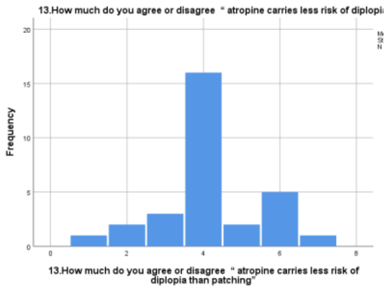

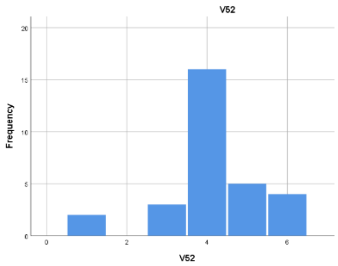

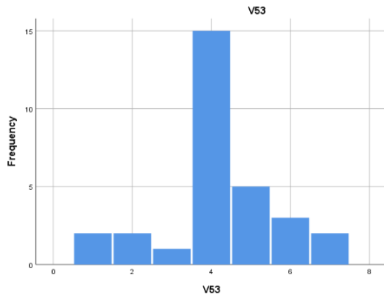

Anisometropia **microtropia with identity** **microtropia without identity**

Question 14

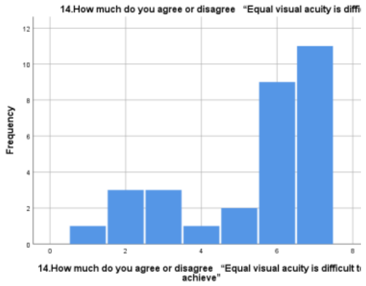

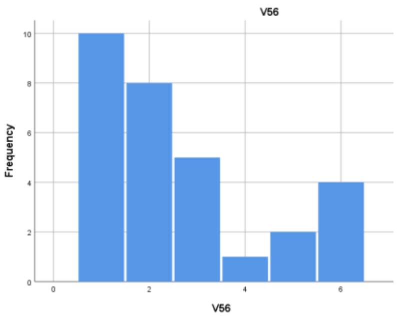

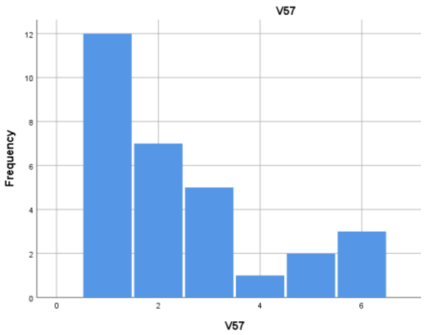

Anisometropia **microtropia with identity** **microtropia without identity**

Question 17

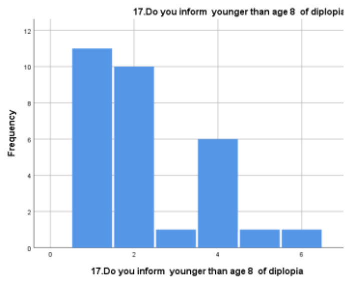

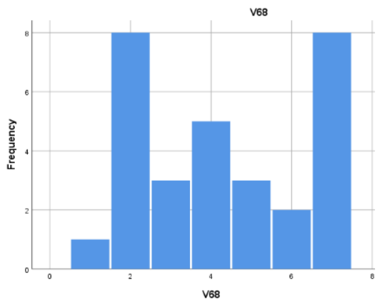

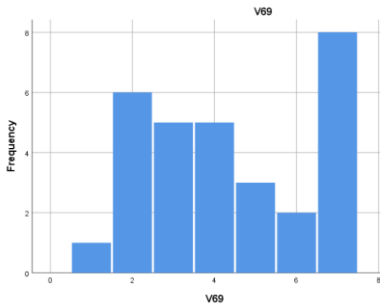

Anisometropia **microtropia with identity** **microtropia without identity**

Question 18

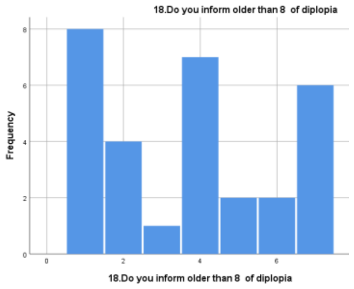

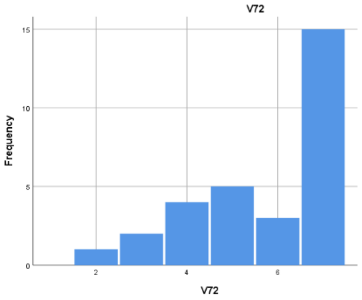

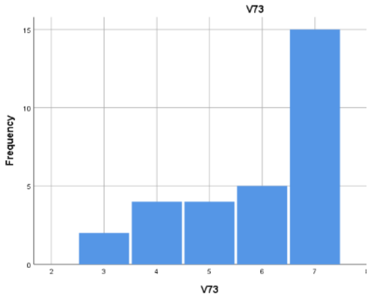

Anisometropia **microtropia with identity** **microtropia without identity**

Question 19

Section 3.2.2

Question 20

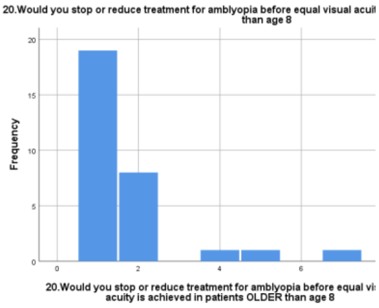

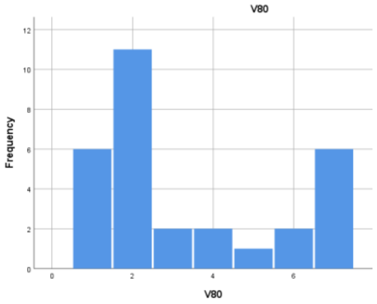

V

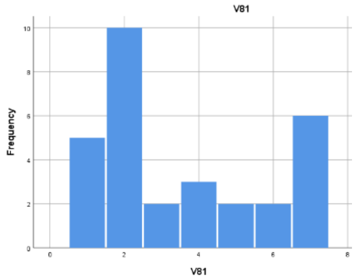

Anisometropia **microtropia with identity** **microtropia without identity**

1.10 Appendix G. Non-parametric analysis

Question 18: Do you inform patients *older than age 8* or parents about the risk of diplopia when treating for amblyopia? Never-always. 1-7

There was a significant difference in how often orthoptists warned families about diplopia in the different conditions. A Friedman test showed a significant difference between conditions ($p<0.001$). A Wilcoxin signed ranks test with Bonferroni correction for three comparisons showed that orthoptists warned families about diplopia significantly more often in microtropia with identity ($p<0.001$) and in microtropia without identity ($p<0.001$) compared to anisometropia. There was no significant difference in how often they warned families about diplopia in microtropia with identity compared to microtropia without identity ($p>0.05$).

Question 19: Would you stop or reduce treatment for amblyopia before equal visual acuity is achieved in patients *younger than age 8* even if the patient has not complained about diplopia and vision is still improving? Never-Always 1-7.

Most orthoptists did not stop amblyopia treatment before equal VA is achieved, but when looking at the distribution of responses (fig 8), a smaller group of orthoptists would almost always stop treatment before equal VA in microtropias.

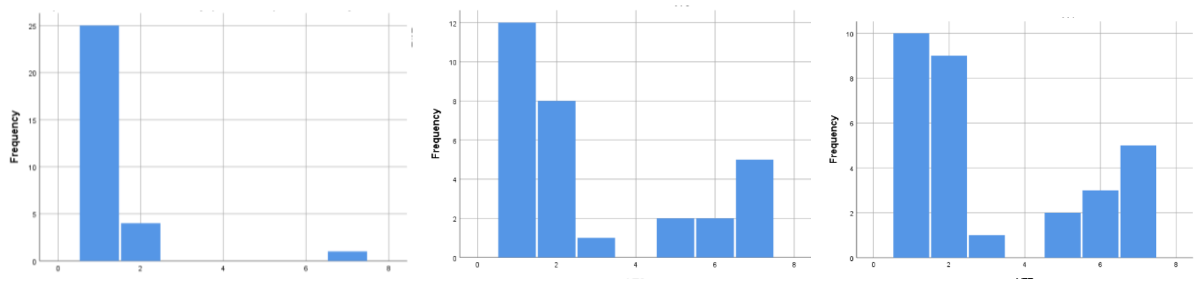

Anisometropia | Microtropia with identity | Microtropia without identity

Histograms showing the frequencies of question 19 of how often orthoptists would stop or reduce amblyopia treatment in the three different conditions in patients younger than age 8.

The histograms showed a spread of distribution of the responses where, even in younger patients, 9 orthoptists would often or always stop treatment in microtropia with identity, and 10 orthoptists would often or always stop treatment in microtropias without identity. Therefore, a Friedman test for non-parametric data was conducted and showed a significant difference between conditions ($p<0.001$). A Wilcoxin signed ranks test with Bonferroni correction for three comparisons showed that orthoptists stopped or reduced treatment significantly more often in microtropia with identity ($p<0.002$) and in microtropia without identity ($p<0.001$) compared to anisometropia. There was no significant difference in how often they stopped or reduced treatment in microtropia with identity compared to microtropia without identity ($p>0.05$).

Question 20: Would you stop or reduce treatment for amblyopia before equal visual acuity is achieved in patients *older than age 8* even if the patient has not complained about diplopia and vision is still improving?

A Friedman test was conducted for patients older than age 8 showing a significant difference between the conditions ($p=0.001$). A Wilcoxin signed ranks test with Bonferroni correction for three comparisons showed that orthoptists stopped or reduced treatment significantly more often in microtropia with identity ($p<0.001$) and in microtropia without identity ($p<0.001$) compared to anisometropia. There was no significant difference in how often they stopped or reduced treatment in microtropia with identity compared to microtropia without identity ($p>0.05$).

The results were in coordination with the ANOVA performed earlier.

Appendix F: Sample size

[1] -- *Monday, May 16, 2022 -- 16:56:49*
F tests - ANOVA: Repeated measures, within factors

**Analysis:**	A priori: Compute required sample size		
**Input:**	Effect size f	=	0.5
	α err prob	=	0.05
	Power (1-β err prob)	=	0.95
	Number of groups	=	1
	Number of measurements	=	3
	Corr among rep measures	=	0.5
	Nonsphericity correction ε	=	1
**Output:**	Noncentrality parameter λ	=	18.0000000
	Critical F	=	3.4433568
	Numerator df	=	2.0000000
	Denominator df	=	22.0000000
	Total sample size	=	12
	Actual power	=	0.9522717

1.11 Appendix H. Differences between countries

Means of questions in table 1,2,9,14,18 and 19 containing only the condition **anisometropia**

	Norway (n=12)	Sweden (n=7)	Denmark (n=4)
How concerned are you for diplopia younger than age 8. Very concerned-not concerned at all 1-7	6.7	6.7	6
How concerned are you for diplopia older than age 8. Very concerned-not concerned at all 1-7	5.1	6	4.5
Stop occlusion before equal VA to prevent diplopia. Very important-not important at all 1-7	5.3	6.4	5
“Equal VA is difficult to achieve” Strongly agree-strongly disagree 1-7.	4.7	5.3	4.5
Stop occlusion even if VA is improving and no diplopia younger than 8. Never-Always 1-7	1.6	1.1	1.3
Stop occlusion even if VA is improving and no diplopia older than 8.	1.9	1.9	2.3

Never-Always 1-7			

Means of questions in table 1,2,9,14,18 and 19 containing only the condition **microtropia without identity**

	Norway (n=12)	Sweden (n=7)	Denmark (n=4)
How concerned are you for diplopia younger than age 8. Very concerned-not concerned at all 1-7	4.6	3.7	4.8
How concerned are you for diplopia older than age 8. Very concerned-not concerned at all 1-7	3.3	2.4	3
Stop occlusion before equal VA to prevent diplopia. Very important-not important at all 1-7	3.6	1.4	3
"Equal VA is difficult to achieve" Strongly agree-strongly disagree 1-7.	2.3	1.6	3
Stop occlusion even if VA is improving and no diplopia younger than 8. Never-Always 1-7	2.4	5.6	2.8
Stop occlusion even if VA is improving and no diplopia older than 8. Never-Always 1-7	3.3	3	5.4

Appendix G: Removal of outlier

One respondent listed the opinion on the other side of the Likert scale than the others. This was repeated on most of the Likert scale questions, giving the impression that the responder interpreted the scale backwards. There was no way to check the responses or making sure the questionnaire had been understood properly. The outlier was therefore withdrawn from the study. The outlier's responses and the group's mean on question 1-10 (Rounded to a whole decimal to clarify the tendency):

The three numbers in the table represent respectively: anisometropia, microtropia with identity, microtropia without identity.

Question	1	2	3	4	5	6	7	8	9	10
The outlier	3,6,7	3,6,7	5,7,7	5,7,7	1,3,7	1,1,2	2,5,7	4,7,7	1,1,5	7,7,7
The group without the outlier (Mean)	7,5,4	6,3,3	6,5,4	6,4,4	3,2,3	3,2,2	5,3,3	5,4,4	6,3,3	6,6,6

Appendix H: Examples of differences in means between countries

Means of questions in table 1,2,9,14,18 and 19 containing only the condition **anisometropia**

	Norway (n=12)	Sweden (n=7)	Denmark (n=4)
How concerned are you for diplopia younger than age 8. Very concerned-not concerned at all 1-7	6.7	6.7	6
How concerned are you for diplopia older than age 8. Very concerned-not concerned at all 1-7	5.1	6	4.5
Stop occlusion before equal VA to prevent diplopia. Very important-not important at all 1-7	5.3	6.4	5
"Equal VA is difficult to achieve" Strongly agree-strongly disagree 1-7.	4.7	5.3	4.5
Stop occlusion even if VA is improving and no diplopia younger than 8. Never-Always 1-7	1.6	1.1	1.3
Stop occlusion even if VA is improving and no diplopia older than 8. Never-Always 1-7	1.9	1.9	2.3

Means of questions in table 1,2,9,14,18 and 19 containing only the condition **microtropia without identity**

	Norway (n=12)	Sweden (n=7)	Denmark (n=4)
How concerned are you for diplopia younger than age 8. Very concerned-not concerned at all 1-7	4.6	3.7	4.8
How concerned are you for diplopia older than age 8. Very concerned-not concerned at all 1-7	3.3	2.4	3
Stop occlusion before equal VA to prevent diplopia. Very important-not important at all 1-7	3.6	1.4	3
"Equal VA is difficult to achieve" Strongly agree-strongly disagree 1-7.	2.3	1.6	3
Stop occlusion even if VA is improving and no diplopia younger than 8. Never-Always 1-7	2.4	5.6	2.8
Stop occlusion even if VA is improving and no diplopia older than 8. Never-Always 1-7	3.3	3	5.4
